# TrkB Overexpression via Gene Therapy: Enhanced Optic Nerve Uptake With Associated Increases in Mitochondria and Axoplasmic Transport

**DOI:** 10.7759/cureus.100594

**Published:** 2026-01-02

**Authors:** Sadat Yazdouni, Andrew Osborne, Keith R Martin

**Affiliations:** 1 School of Clinical Medicine, University of Cambridge, Cambridge, GBR; 2 Department of Medicine, Imperial College Healthcare NHS Trust, London, GBR; 3 John van Geest Centre for Brain Repair, Department of Clinical Neurosciences, University of Cambridge, Cambridge, GBR; 4 Ocular Gene Therapy, Ikarovec Ltd, The Norwich Research Park Innovation Centre, Norwich, GBR; 5 Department of Ophthalmology, Centre for Eye Research Australia, Melbourne, AUS; 6 Department of Ophthalmology, Addenbrooke’s Hospital, Cambridge, GBR; 7 Department of Surgery/Ophthalmology, University of Melbourne, Melbourne, AUS; 8 Department of Ophthalmology, Royal Victorian Eye and Ear Hospital, Melbourne, AUS

**Keywords:** adeno-associated viral vector, axoplasmic transport, brain-derived neurotrophic factor (bdnf), glaucoma, ocular gene therapy, rodent model, tropomyosin-related receptor kinase-b

## Abstract

Introduction: Glaucoma is a progressive neurodegenerative disease that affects retinal ganglion cells (RGCs), ultimately leading to vision loss. In this study, we investigated gene therapy-mediated transduction of RGCs and examined axonal transport changes in the optic nerve using a viral vector designed to upregulate tropomyosin receptor kinase B (TrkB) expression.

Method: TrkB expression was evaluated in retinae and optic nerves of rats following genetic intravitreal delivery of AAV2-TrkB. Axonal transport and preliminary mitochondrial changes were assessed in optic nerves by immunohistochemical staining for kinesin and voltage-dependent anion channel (VDAC), a mitochondrial component.

Results: The results revealed an approximately 30% increase in TrkB expression in the retina, which was confirmed to be vector-driven by a P2A tag attached to the TrkB protein. This increased protein expression could be seen independent of injury and in eyes with elevated intraocular pressure. Observations along the optic nerve of rats treated with AAV2-TrkB revealed elevated transport of TrkB along axons (50% in TrkB, 120% in P2A tag) and significant increases in kinesin (12%) and VDAC (16%) immunoreactivity.

Conclusion: This study provides early indications that improving TrkB expression in the eye may increase anterograde transport of motor proteins, which in turn could improve mitochondrial transport within the optic nerve.

## Introduction

Glaucoma is a leading cause of irreversible blindness worldwide [1]. Clinically, glaucoma is characterised by distinguishable ‘cupping’ of the optic nerve head (ONH), thinning of the peripapillary retinal nerve fibre layer and retinal ganglion cell (RGC) death, leading to subsequent vision loss. Axons, which relay information from the retina to the brain, pass through the ONH and are thought to undergo axonal cytoskeletal changes, likely due to compression of the nerve bundle and supporting vasculature. This compression is contemporaneous with significant axonal transport reduction [2].

The axons of RGCs are limited in their ability to synthesise proteins and other molecules that are important for their maintenance and function. Distal ends of RGC axons in the brain are relatively far away from their cell bodies in the retina; therefore, newly synthesised proteins, organelles, and other synaptic components from the cell bodies are anterogradely transported (towards the brain) by attaching to motor proteins, kinesins [3]. Damaged organelles and proteins from the distal axon are retrogradely transported back to the soma for degradation and recycling via a different family of motor proteins, dyneins [3]. The anterograde and retrograde transport of neurotrophins is important for cell response to injury and growth, with both motor proteins dependent on ATP hydrolysis to generate the energy needed to move along the cytoskeleton [4].

RGCs and their axons require vast amounts of energy for their function and survival. The mechanisms of dynamic instability of microtubules, which motor proteins use to travel along, also require energy. Therefore, insufficiencies in the transport of mitochondria to sustain the ATP demands of the axons can provoke global neuronal malfunction.

Studies have suggested that elevated intraocular pressure (IOP) disrupts mitochondrial transport by kinesin within the optic nerve, leading to reduced ATP levels, increased reactive oxygen species, and apoptosis, which collectively contribute to RGC death [5-9]. Early gene therapy studies targeting oxidative stress, such as supplementation of the superoxide dismutase 2 (*SOD2*) gene and disrupted in schizophrenia 1 (DISC1) protein, show promise in restoring mitochondrial transport and reducing neurodegeneration [10,11], highlighting axonal transport as a critical, potentially reversible therapeutic target.

Alterations in tropomyosin receptor kinase B (TrkB) receptor and its ligand, brain-derived neurotrophic factor (BDNF), have been noted in glaucoma. BDNF binding of TrkB receptor can initiate the activation of molecular signalling cascades associated with neuroprotection, neurogenesis, axon growth [12], as well as improved axoplasmic transport [13] and mitochondrial transport [14]. However, TrkB receptor expression has been shown to be downregulated after optic nerve injury [15,16]. This provided the rationale for this TrkB supplementation study and highlights the importance of understanding mitochondrial dysfunction and disruptions in axoplasmic transport in the complex pathogenesis of glaucoma.

The primary aim of this study was to determine whether intravitreal adeno-associated virus serotype 2 (AAV2)-mediated TrkB overexpression enhances anterograde axonal transport in retinal ganglion cells under normotensive and ocular hypertensive conditions. Our secondary aims were to assess whether increased TrkB expression is associated with elevated levels of transport-related proteins, including kinesin and voltage-dependent anion channel (VDAC), within the optic nerve. We hypothesised that TrkB upregulation would promote improved axoplasmic and mitochondrial transport, thereby addressing early pathological mechanisms in glaucoma.

## Materials and methods

AAV2 vectors

An AAV2 coding for TrkB was created with a P2A tag (GSG)ATNFSLLKQAGDVEENPGP attached to the C-terminus of TrkB, where it would be expressed intracellularly on transduced cells (Charles River (formerly, Vigene Biosciences), Rockville, Maryland, United States). A viral P2A tag was selected over a His tag due to its translational potential and the anticipation that the P2A would aid visualisation of the translated protein above endogenous TrkB expression. A Null vector containing no transgene was used for comparison.

Research animals

Twenty adult male Sprague-Dawley rats (150g; Charles River) were used for the laser induced ocular hypertension model of experimental glaucoma and to assess expression within the eye and optic nerve. All experiments were carried out in accordance with the United Kingdom (UK) Home Office Regulations for the Care and Use of Laboratory Animals, the UK Animals (Scientific Procedures) Act 1986, and the Association for Research in Vision and Ophthalmology’s Statement for the Use of Animals in Ophthalmic and Visual Research. This was approved by the University of Cambridge Animal Ethics Committee (Project Licence 70/8152).

Rat injections and glaucoma modelling

Animals were randomly assigned to receive either AAV2-TrkB or AAV2-Null injections using a simple randomisation procedure. Sample sizes were based on prior studies using comparable rat glaucoma models and AAV2 vectors, which have demonstrated robust detectable differences in protein expression and axonal transport [17].

The left eye of animals was lasered using an established model to elevate IOP over 40 days [17]. Contralateral fellow eyes served as normotensive controls and showed no elevation of pressure during regular IOP measurements. The IOP model was selected as it’s been shown to cause gradual RGC death [18], much like the natural progression of glaucoma in humans.

Three weeks prior to the laser procedure, both eyes of rats were injected with AAV2-TrkB or AAV2-Null at a titre of 5E9 genome copies/eye. Four weeks post IOP elevation, seven weeks after the initial intravitreal injection, eyes and nerves were collected and evaluated for expression, evidence of transport, and mitochondrial protein upregulation. Researchers conducting the downstream experiments were blinded throughout the staining, imaging, and analysis process.

Immunohistochemistry

Following transcardial perfusion with 4% paraformaldehyde (PFA) (EMD Millipore, Inc., Burlington, Massachusetts, United States) under terminal anaesthesia, the eyes and nerves of rats were collected and further fixed for 24 hours in 4% PFA. Eyes and nerves were then dehydrated in 30% sucrose (Thermo Fisher Scientific Inc., Waltham, Massachusetts, United States) and phosphate-buffered saline (PBS) (Gibco; Thermo Fisher Scientific Inc.) at 4°C for 24 hours. The retinae and nerves were then carefully placed in small silicon microtubes filled with optimal cutting temperature gel (Tissue-TEK; Sakura Finetek Japan Co., Ltd., Tokyo, Japan). The silicon tubes were frozen on dry ice, and retinae sectioned at 13 μm on a Bright OTF5000 Cryostat (Bright Instrument Co. Limited, Huntingdon, United Kingdom) and mounted onto superfrost plus slides (VWR International, Radnor, Pennsylvania, United States). Optic nerves were sectioned at a thickness of 20 µm as detailed before [17].

Slides were submerged in PBS and placed on a rocker shaker for 15 minutes. Following an additional two washes, tissue sections were permeabilised and blocked with 500 µL blocking buffer (PBS/2% bovine serum albumin/0.3% Triton X-100/5% normal goat serum) for one hour at room temperature in the absence of light. Retina or optic nerve tissue was then rinsed and incubated overnight at 4°C in 500 µL of primary antibody diluted in the blocking buffer (Table 1).

**Table 1:** Primary and secondary antibodies used for immunohistochemical staining

Antibody	Supplier	Catalogue Number	Antibody dilution
Primary Antibody			
Rabbit TrkB antibody	Abcam, part of Danaher Corporation, Washington, D.C., United States	AB33655	0.20%
Mouse P2A antibody	Novus Biologicals LLC, Centennial, Colorado, United States	NBP2-59627	0.20%
Mouse VDAC antibody	Abcam, part of Danaher Corporation, Washington, D.C., United States	AB14734	0.20%
Rabbit Kinesin antibody	Abcam, part of Danaher Corporation, Washington, D.C., United States	AB62104	0.20%
Secondary Antibody			
Goat anti-rabbit – 488	Thermo Fisher Scientific Inc., Waltham, Massachusetts, United States	A11034	0.10%
Goat anti-mouse – 555	Thermo Fisher Scientific Inc., Waltham, Massachusetts, United States	A21424	0.10%
Goat anti-mouse – 647	Thermo Fisher Scientific Inc., Waltham, Massachusetts, United States	A21235	0.10%
Goat anti-rabbit – 647	Thermo Fisher Scientific Inc., Waltham, Massachusetts, United States	A32733	0.10%
DAPI	Thermo Fisher Scientific Inc., Waltham, Massachusetts, United States	1217826	0.10%

Slides were rinsed in PBS three times, 15 minutes each, and 500 µL of secondary antibody diluted in the blocking buffer (Table 1), added at room temperature in the absence of light, for two hours. Slides were rinsed in PBS again (3 x 15 minutes) and mounted ready for imaging.

An SP5 confocal microscope (Leica Microsystems, Wetzlar, Germany) was used to image the sections at x40 magnification with overview images at x20 magnification. Three suitable sections of the same retinal sample or optic nerve were imaged and processed using ImageJ (1.8.0_172; imagej.net).

Image analysis

For retinal analysis, the images were stacked to maximum intensity and rotated so that the RGC layer was orientated to the left of the screen. A box was drawn from the start of the GCL to the end of the outer nuclear layer (ONL), and a plot profile of fluorescence intensity for 4′,6-diamidino-2-phenylindole (DAPI), TrkB, and 2A was generated. Area under the curve (AUC) values were then measured for each sample and grouped.

All images were quantified using consistent regions of interest of fixed dimensions across samples. Thresholding for fluorescence detection was applied uniformly using ImageJ’s automatic thresholding functions, and no manual adjustment was made between samples. Imaging parameters, including laser intensity, gain, and exposure time, were held constant for all specimens within each staining set to ensure comparability. All analyses were performed using the same software version (ImageJ 1.8.0_172) to ensure consistency in processing and quantification.

AUC calculation

The trapezoidal rule was used to calculate the AUC for the graph plotting x = depth through the retinal layers, y= immunofluorescence intensity. The trapezoidal rule is a mathematical technique used to approximate the definite integral (exact AUC). 

$\int_a^b f(x)\,dx$

The method works by approximating the area under the function f(x) as a trapezoid, calculating the area of the trapezoids and adding them together. It follows that:

$\int_a^b f(x)\,dx \approx (b - a)\,\frac{f(a) + f(b)}{2}$

**Figure 1:**
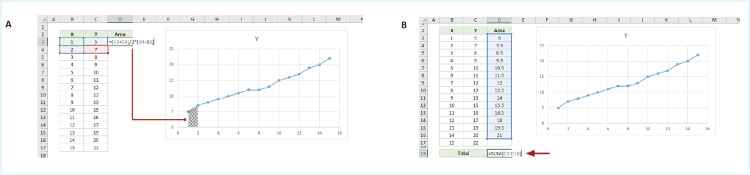
Area under the curve (AUC) calculation A. Screenshot of a Microsoft Excel (Microsoft Corporation, Washington, United States) sheet showing a formula input to work out AUC of the trapezoid shaded. B. Screenshot of a Microsoft Excel sheet showing the sum of all trapezoid AUCs.

By this logic, areas of trapezoids between x=1 and x=2 (Figure 1A) can be calculated with the following formula on Excel (Microsoft Corporation, Washington, United States): =(C3+C4)/2*(B4-B3). The AUC values were grouped using GraphPad Prism (8.2.1.441; Dotmatics, Boston, Massachusetts, United States), and an unpaired, two-tailed t-test was used to compare AAV2-TrkB to AAV2-Null groups.

Optic nerve imaging and analysis

Fluorescence intensity quantification within optic nerves was carried out for TrkB, P2A, VDAC, and kinesin. A fixed-size area was used for each image, and a mean fluorescence intensity was calculated in ImageJ. Significance was set at 0.05, and an unpaired, two-tailed t-test was used to compare AAV2-TrkB to AAV2-Null groups, with individual data points shown, each data point representing a uniquely treated eye.

## Results

Confocal imaging of retinal TrkB and P2A expression

**Figure 2:**
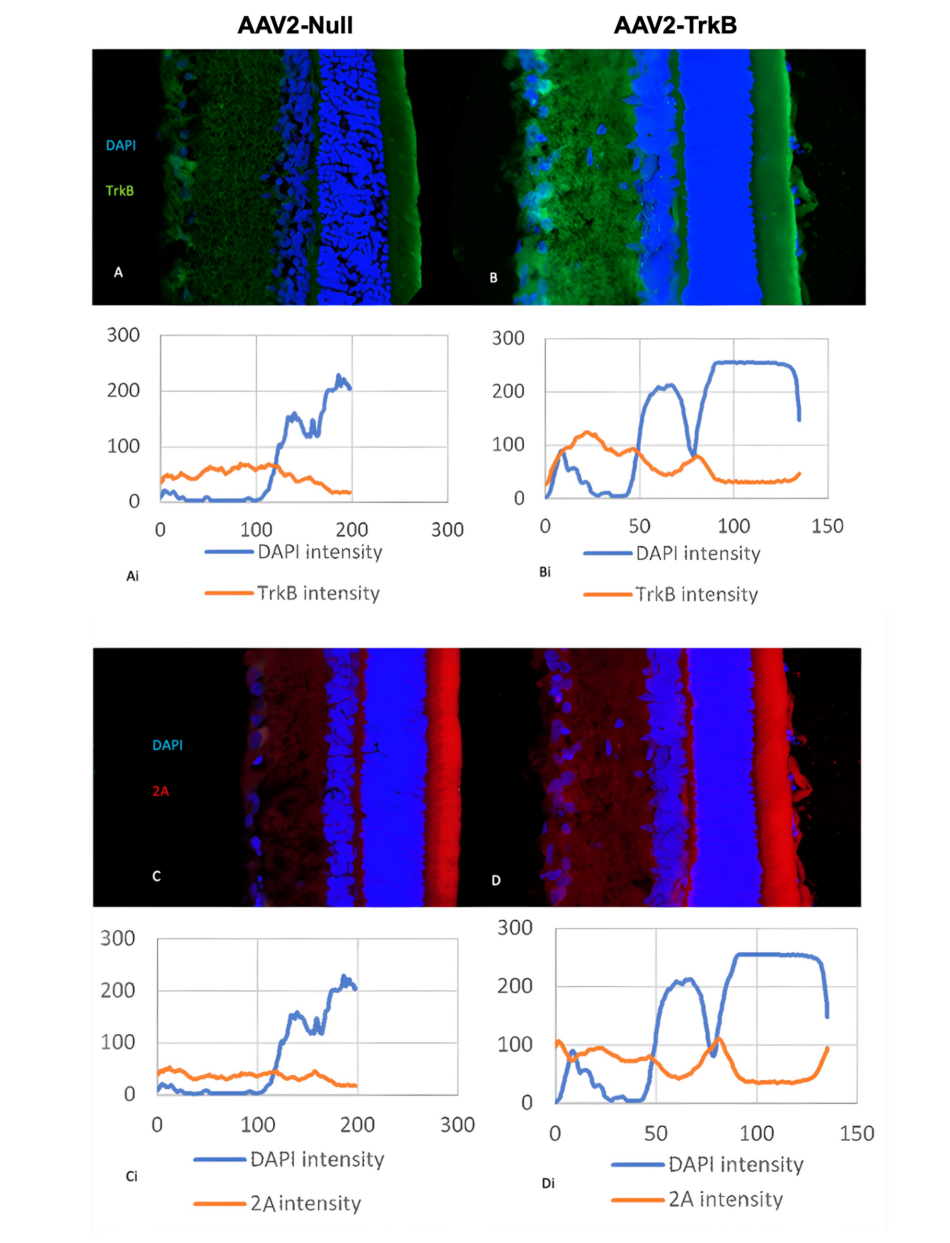
Confocal imaging of rat retinal sections treated with AAV2-Null (left) vs AAV2-TrkB (right) and corresponding immunofluorescence intensity graphs A-D: Confocal images of rat retinal sections showing DAPI (blue), TrkB (green), and P2A (red) following injection with AAV2-Null (left) or AAV2-TrkB (right). Panels A and B show TrkB labelling, while panels C and D show P2A labelling indicating vector-driven TrkB expression. Ai-Di: Corresponding fluorescence intensity line profiles for each section. The graphs show DAPI intensity relative to TrkB (Ai, Bi) or P2A (Ci, Di) across retinal depth, demonstrating increased TrkB and P2A signal in AAV2-TrkB-treated eyes.

Confocal imaging of retinal sections (Figure 2) further illustrates the distribution of TrkB and P2A labelling in relation to retinal structure. DAPI staining was used to identify and outline the nuclear layers, allowing clear visualisation of TrkB and P2A localisation across retinal depth. In AAV2-TrkB-treated eyes, TrkB immunoreactivity was visibly increased throughout the retinal layers, particularly within the ganglion cell layer, compared with AAV2-Null controls (Figure 2A-2B). Similarly, P2A labelling, which marks vector-derived TrkB expression, showed stronger signal intensity in the AAV2-TrkB group (Figure 2C-2D). The corresponding fluorescence intensity profiles (Figure 2Ai-2Di) demonstrate higher TrkB and P2A signal across retinal depth in AAV2-TrkB-treated eyes, supporting the quantitative increases reported below.

TrkB and P2A immunoreactivity in normotensive rat retinae

**Figure 3:**
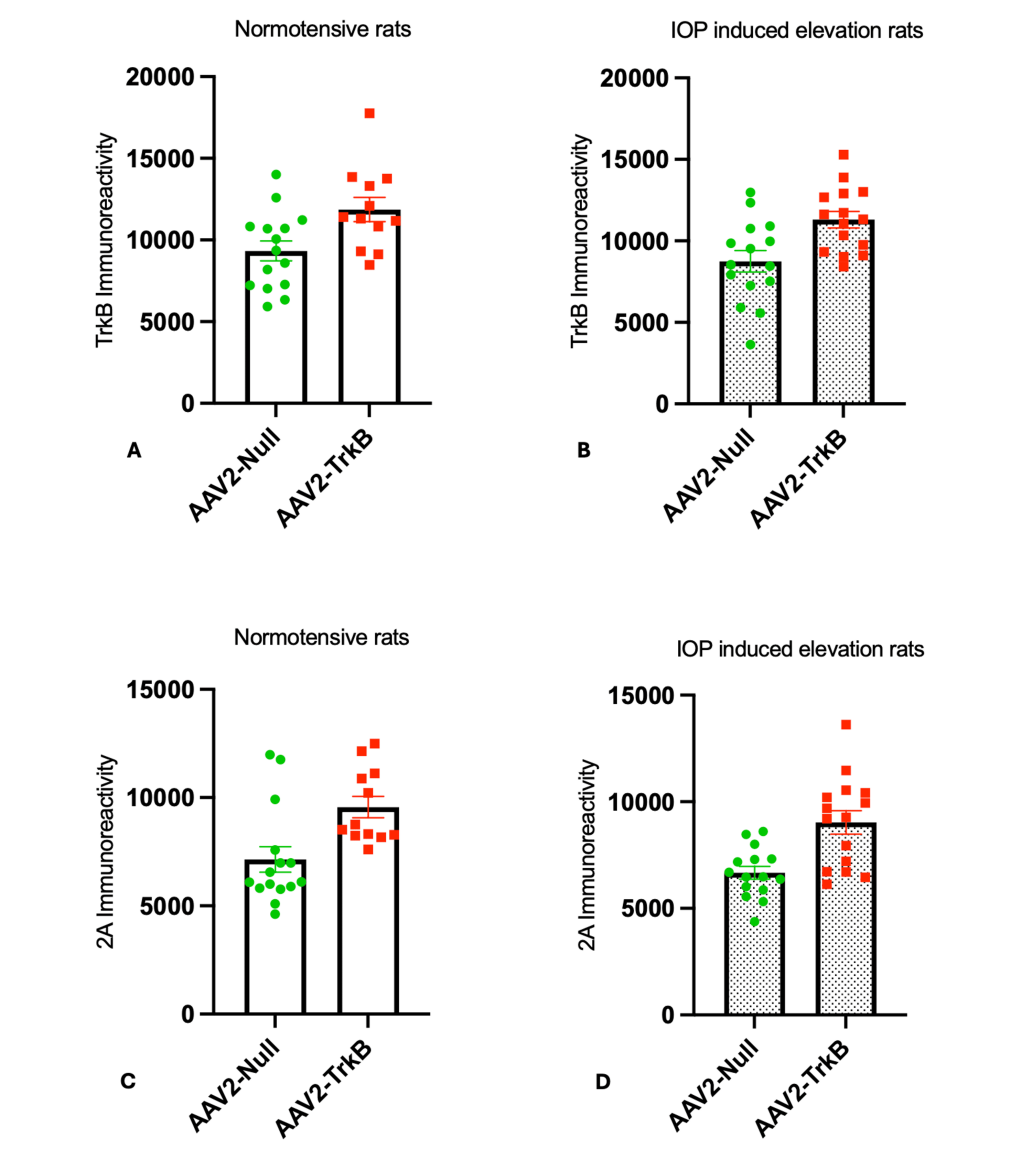
Immunoreactivity of TrkB and P2A in rat retinae given: AAV2-Null and AAV2-TrkB A–B: TrkB immunoreactivity in normotensive (A) and IOP-elevated (B) rat retinae following AAV2-Null or AAV2-TrkB injection. TrkB expression was significantly higher in AAV2-TrkB–treated eyes under both conditions. C–D: P2A immunoreactivity in normotensive (C) and IOP-elevated (D) retinae, confirming vector-driven TrkB overexpression. AAV2-TrkB–treated eyes showed significantly increased P2A signal compared with AAV2-Null controls. Statistical values: A: AAV2-TrkB 11,864 ± 738.9 vs. AAV2-Null 9,333 ± 613 (t=2.660, df=25, p < 0.05) B: AAV2-TrkB 11,300 ± 515.7 vs. AAV2-Null 8,746 ± 660.8 (t=3.047, df=28, p < 0.01) C: AAV2-TrkB 9,561 ± 494.7 vs. AAV2-Null 7,114 ± 586.5 (t=3.053, df=25, p < 0.01) D: AAV2-TrkB 9,034 ± 553.2 vs. AAV2-Null 6,669 ± 303.5 (t=3.749, df=28, p < 0.001) IOP: intraocular pressure

TrkB and P2A expression was examined in immunostained sections of retinae from rats seven weeks post intravitreal injections of AAV2 vectors. An almost 30% increase in immunoreactivity of TrkB was observed throughout the retina, predominantly in the GCL of eyes injected with AAV2-TrkB compared to AAV2-Null (AAV2-TrkB = 11864 ± 738.9, AAV2-Null = 9333 ± 613, p < 0.05; Figure 3A). Similarly, the viral P2A tag bound to newly synthesised TrkB receptors was increased in the retina of rat eyes injected with AAV2-TrkB, which showed a 30% increase in P2A immunoreactivity when compared to eyes treated with AAV2-Null (AAV2-TrkB = 9561 ± 494.7, AAV2-Null = 7114 ± 586.5, p < 0.01; Figure 3C). 

TrkB and P2A immunoreactivity in ocular hypertensive rat retinae

TrkB and P2A immunoreactivity were also elevated in the retinae of rat eyes subjected to induced ocular hypertension. Similar to uninjured contralateral eyes, ocular hypertensive eyes injected with AAV2-TrkB had an approximately 30% increase in TrkB immunoreactivity when compared to controls (AAV2-TrkB = 11300 ± 515.7, AAV2-Null = 8746 ± 660.8, p < 0.01; Figure 3B). A similar increase was observed for P2A (AAV2-TrkB = 9034 ± 553.2, AAV2-Null = 6669 ± 303.5, p < 0.001; Figure 3D). 

TrkB and P2A detection in the optic nerve

**Figure 4:**
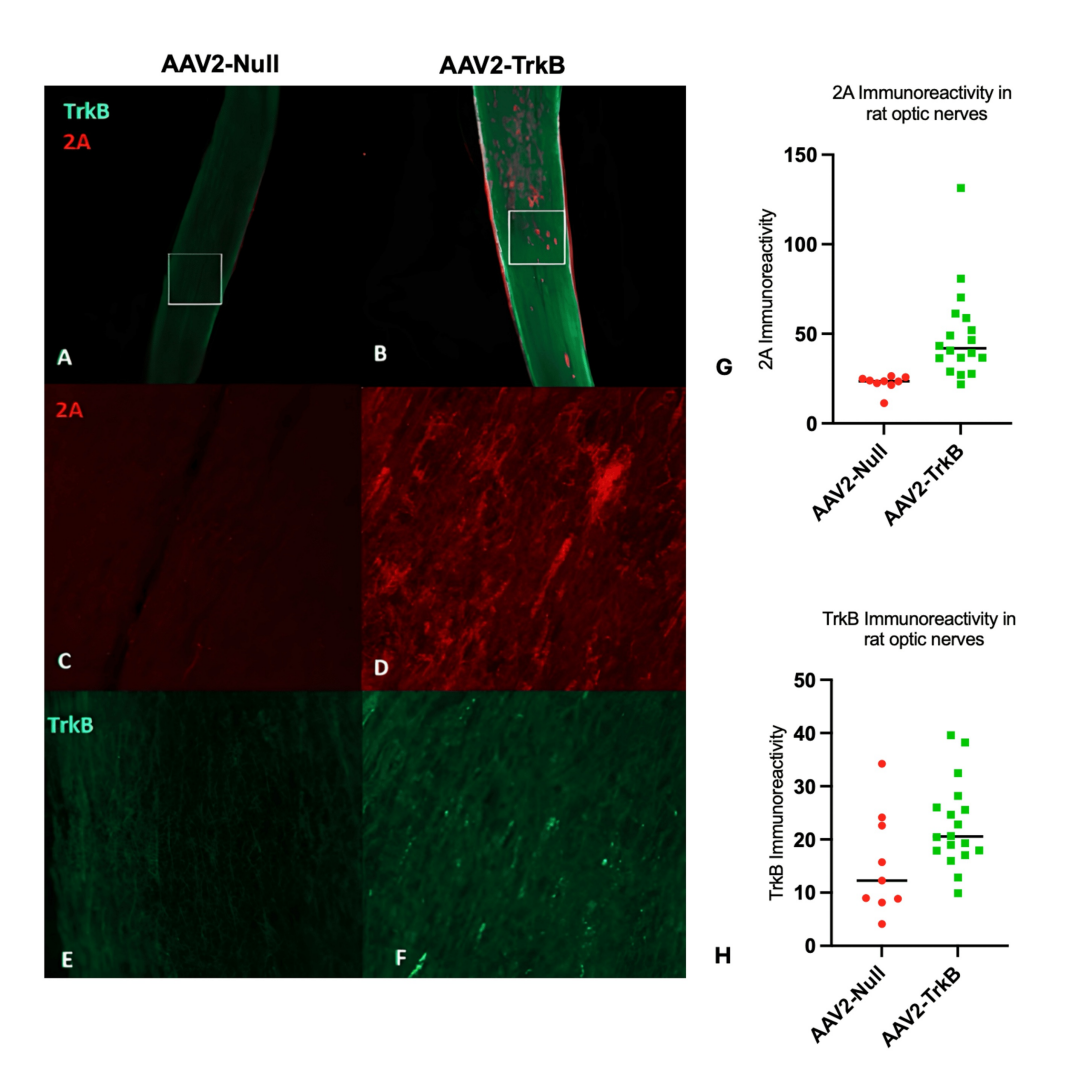
Epifluorescence imaging and quantification of TrkB and P2A immunoreactivity in rat optic nerves following AAV2-Null or AAV2-TrkB treatment A-F: Epifluorescence images of rat optic nerves labelled for P2A and TrkB following AAV2-Null (A, C, E) or AAV2-TrkB (B, D, F) injection. Panels A and B show whole-nerve overviews, with boxed regions indicating areas used for higher-magnification confocal imaging shown in C-F. P2A signal is shown in C-D, and TrkB signal is shown in E-F. G-H: Quantification of P2A (G) and TrkB (H) immunoreactivity in optic nerves. Both markers showed significantly higher fluorescence intensity in AAV2-TrkB–treated nerves compared with AAV2-Null controls. Statistical values: G: AAV2-TrkB = 49.37 ± 6.07, AAV2-Null 22.58 ± 1.51 (t = 3.069, df = 25, p < 0.01). H: AAV2-TrkB = 22.69 ± 1.891, AAV2-Null = 15.45 ± 3.246 (t = 2.060, df = 25, p < 0.05).

P2A and TrkB expression were also explored along the optic nerve of rat eyes injected with either AAV2-TrkB or AAV2-Null (Figure 4A-4F). An almost 50% increase in TrkB immunoreactivity was observed along optic nerve sections in eyes treated with AAV2-TrkB compared to the control (AAV2-TrkB = 22.69 ± 1.891, AAV2-Null = 15.45 ± 3.246, p < 0.05; Figure 4H) with viral P2A immunoreactivity, which was clearer to detect due to low endogenous expression, highlighting an almost 120% increase in detection along the nerve in AAV2-TrkB groups compared to AAV2-Null (AAV2-TrkB = 49.37 ± 6.067, AAV2-Null = 22.58 ± 1.507, p < 0.01; Figure 4G).

Mitochondrial transport in the optic nerve after intravitreal AAV2 TrkB therapy

**Figure 5:**
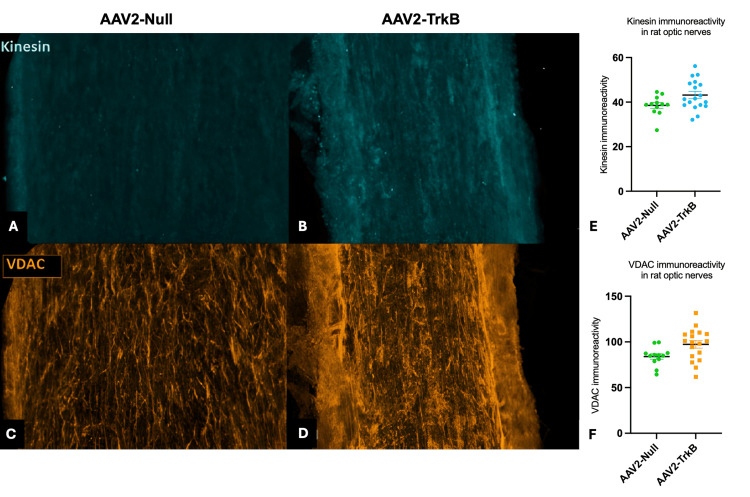
Confocal imaging and immunoreactivity of kinesin and VDAC in rat optic nerves following AAV2-Null or AAV2-TrkB treatment A-D: Confocal images of kinesin (A-B) and VDAC (C-D) immunoreactivity in rat optic nerves following AAV2-Null (A, C) or AAV2-TrkB (B, D) treatment. Increased signal intensity for both kinesin and VDAC is evident in AAV2-TrkB-treated nerves compared with AAV2-Null controls. E-F: Quantification of kinesin (E) and VDAC (F) immunoreactivity. AAV2-TrkB-treated optic nerves showed significantly higher fluorescence intensity for both markers than AAV2-Null. Statistical values: E: AAV2-TrkB 43.16 ± 1.58 vs. AAV2-Null 38.51 ± 1.29 (t = 2.109, df = 28, p < 0.05) F: AAV2-TrkB 97.29 ± 4.11 vs. AAV2-Null 83.96 ± 2.96 (t = 2.379, df = 28, p < 0.05) VDAC: voltage-dependent anion channel

Immunohistochemistry using antibodies targeting kinesin, an axonal transport protein, was then performed to see the impact AAV2-TrkB may be having on axonal transport (Figure 5A-5B). AAV2-TrkB-treated eyes had a 12% increase in axonal immunoreactivity of kinesin when compared to controls (AAV2-TrkB = 43.16 ± 1.576, AAV2-Null = 38.51 ± 1.292, p < 0.05; Figure 5E). VDAC, a mitochondrial component, also showed elevated detection in optic nerve sections of rat eyes treated with AAV2-TrkB (Figure 5C-5D), with expression increased 16% over controls (AAV2-TrkB = 97.29 ± 4.112, AAV2-Null = 83.96 ± 2.956, p < 0.05; Figure 5F).

## Discussion

There is growing evidence to suggest that a reduction in BDNF and TrkB signalling is a key attribute in the progression of glaucoma [19,20]. BDNF supplementation has been shown to be neuroprotective and minimises disease progression; however, sustained protection has been limited, in part, due to downregulation of its receptor, TrkB [21]. Reduced optic nerve axonal transport is also thought to be responsible for glaucoma progression. We showed in 2021 that overexpression of TrkB and BDNF could improve axonal transport along the optic nerve [22]. Here, we provide supportive evidence that enhanced TrkB expression within RGCs via gene therapy correlates with elevated proteins that could improve mitochondrial transport along the optic nerve.

This study also provides an early indication that a viral P2A tag to a protein, which is not native to humans, can aid the identification of upregulated proteins in the retina and optic nerve. To that effect, the presence of the P2A protein would suggest that the increase of TrkB receptor expression in treated specimens is concomitant with treatment, and P2A could serve as a useful tag in future testing.

Axonal transport disruptions have been shown to precede RGC death in animal studies [23]. By restoring axonal transport early in the chronology of glaucoma progression, studies were able to prevent a considerable amount of RGC death and even help regenerate axons that were previously damaged [24]. Finding mechanisms of axonal transport rescue may shed light on possible therapeutics that could work synergistically with IOP-lowering treatments, especially for patients who have continued glaucoma progression despite IOP-lowering treatment.

In particular, kinesins are quintessential proteins responsible for the anterograde transport of molecules, organelles, lipids, and proteins along the nerve axon [3]. Studies on rat models of glaucoma have revealed marked reduction of kinesin proteins in the optic nerve [25], and it is well documented that axonal transport deficits are one of the early physiological changes associated with glaucoma pathology [26] with deficits in anterograde transport, facilitated by kinesin proteins, preceding retrograde transport deficits in DBA/2J mouse models of glaucoma [27]. This would suggest that kinesin proteins are affected very early on in the chronology of glaucoma progression and that early rescue of kinesin functionality could prevent downstream neuronal dysfunction.

Our research demonstrates that gene therapy for a known modulator of axonal transport potentially improves transport along axons of the optic nerves via the increase in kinesin proteins. Additionally, the improved axon transport may be responsible for the increased TrkB anterograde transport from the RGC body along the axon. Whether this transport in the optic nerve is beneficial for the survival and repair of the axon remains to be seen, but the principle that motor protein transport is elevated could lead to possible other regenerative molecules being transported to the damaged axon tip.

Retinal cells and their axons require vast amounts of energy to function [28]. Axons within the optic nerve are dependent on a constant supply of ATP to carry out their functions of relaying action potentials, transporting molecules on motor proteins, and maintaining the cytoskeleton on which transport molecules use to travel [29]. Therefore, it comes as little surprise that studies have also shown that mitochondrial depletion in the optic nerve may be associated with glaucoma [30] and that mitochondria-preserving therapies may be useful in preventing optic nerve degeneration and subsequent progression to irreversible glaucomatous vision loss.

This study showed a modest increase in immunoreactivity of VDAC, a protein located on the outer mitochondrial membrane and a surrogate marker of mitochondria, in the optic nerve of AAV2-TrkB-injected eyes. It may therefore be possible that the increase in VDAC is also associated with an increase in mitochondria in the optic nerve. The logic goes that: more mitochondria would be able to produce more ATP, which is imperative for axonal function and survival; therefore, more axons would be able to survive and potentially regenerate. An increase in mitochondria may suggest a further beneficial mechanism of neuroprotection associated with this gene therapy.

Limitations of the study

This study has several limitations. The sample size was modest and restricted to a single rat strain, which may limit the generalisability of the findings. Immunofluorescence intensity was used as a surrogate measure of protein expression and of axonal or mitochondrial transport; although informative, it does not directly assess transport kinetics or mitochondrial motility. Accordingly, future studies incorporating live imaging or dedicated axonal transport assays would provide more definitive functional insight. Similarly, while increased kinesin and VDAC immunoreactivity suggest potential enhancement of axonal and mitochondrial transport, these findings require confirmation through electrophysiological or other functional outcome measures.

AAV2-TrkB treatment was administered prior to laser-induced ocular hypertension. This design allowed us to assess whether early TrkB expression could preserve axonal transport capacity under glaucomatous stress; however, it did not evaluate post-injury intervention, which remains an important clinical consideration. Addressing the therapeutic efficacy of TrkB gene delivery after injury will require a separate study with additional experimental groups and extended timelines.

In addition, ATP concentrations and mitochondrial bioenergetic parameters were not quantified. Direct quantification of ATP or mitochondrial bioenergetics was beyond the scope of this preliminary work but would strengthen future investigations. Finally, although the P2A tag facilitated detection of vector-derived TrkB, subtle effects of the tag on protein localisation or stability cannot be entirely excluded.

## Conclusions

Current treatments for glaucoma primarily aim to lower IOP; however, some patients continue to experience disease progression despite IOP-lowering therapies. Disruptions in axoplasmic transport, implicated in the early pathogenesis of glaucoma, represent a potentially reversible therapeutic target.

This study confirms AAV2-TrkB is capable of transducing RGCs in rats, and TrkB and 2A expression can be detected in the optic nerves. We demonstrated that there was an increase in anterograde transport of motor proteins, which could improve mitochondrial transport. Therefore, there is a rationale to further investigate the mechanisms behind improved axonal transport with AAV2-TrkB and the potential role mitochondria have in regeneration.
